# The First Permanent Molar

**Published:** 1890-08

**Authors:** J. F. Colyer


ARTICLE V.

### THE FIRST PERMANENT MOLAR.

BY J. F. COLYER, M.R.C.S., L.R.C.P., L.D.S.

Probably no tooth in the whole dental arch has given rise to more discussion as to its treatment than that of the first permanent molar, and in the following remarks no new idea has been advanced, but the question of treatment has been briefly reviewed. The six-year old molar is of special interest to us from more than one point of view.

Physiologically it is the most useful tooth in the whole arch, since it presents the largest area of crown surface, is situate in a position where mastication is greatest, and is admirably adapted to bear the strain thus put upon it, inserted as it is into the molar process, the thickest portion of the maxillary bone.

Pathologically, the first permanent molar is of interest, since it is more liable to caries than any other tooth, caused probably by its structure being weakened in its development at a period when nutrition in the child is often at fault.

The six-year olds are also of great importance in preserving the integrity of the arch, since should they be lost prior to the eruption of the second permanent molars, irregularity may frequently be caused in the other teeth ; or if later than this period, the second bicuspid and second molar will constantly tilt towards each other, and thus lose their use in mastication to some extent, or cause disarrangement of the bite.

From these facts it is evident that our decisions on a course of treatment in these cases will at times be a matter of the utmost difficulty.

In the six-year old then, we have these two great characteristics, the one militating against the other, *idem est* :—

its physiological utility in mastication and its pathological tendency to decay. Presuming these to be set off against each other, our treatment will almost invariably turn upon the question of irregularity.

Probably the best method of treating the subject will be to assume the various cases that most frequently come before our notice and discuss them separately.

(i.) *Given an overcrowded mouth and moderately sound six-year olds.*—There can be no question of our treatment in these cases, the bicuspids should be removed, and the molar, a tooth of far greater importance in mastication, should be saved. The same treatment will apply when a slight cavity may exist in the six-year old molars.

Some practitioners always make it a rule to remove the six-year olds rather than the bicuspids on account of the greater liability to decay in the former. We would ask them to remember that the molar has withstood the secretions of the mouth for a much longer period than the bicuspid, and hence there is no reason why it should be a weaker tooth. We may even lay it down as a rule, that a six-year old molar remaining healthy until the age of twelve is far less prone to approximal decay than the bicuspid.

(ii.) *Given an overcrowded mouth and carious six-year olds.*—There can be no doubt that in these cases we should adopt radical treatment and not only should we extract such molars as are carious but we strenuously advise the removal of all four six-year olds. Nature will usually speedily overcome any crowding, and the gaps left will be gradually filled by the twelve-year old molars and second bicuspids. The travelling backwards of the bicuspids is brought about in the following manner: each aspect of the crown surface of these teeth presents practically an inclined plane. Now these antagonistic planes will naturally act upon one another in the direction of least resistance, and should the four six-year olds have been extracted as suggested, we get the bicuspids moving backwards to at least partially occupy the spaces left.

Should only one first permanent molar be extracted, the opposing teeth in the other jaw will be locked, we shall fail to get any natural backward action, and shall have to resort to artificial means in order to pull back the bicuspids. I need scarcely mention here that mechanical contrivances should always be avoided if possible. Again, should only one six-year old be extracted, the corresponding tooth in the other jaw is to all intents and purposes useless in mastication.

Hence with an overcrowded mouth and carious six-year olds, we strongly advise the extraction of all four.

(iii.) *Given a case with practically no overcrowding and carious six-year olds.*—The amount of decay that has taken place, and the number of six-year olds attacked by caries, must be taken into account. It is very unusual to find one of the six-year olds carious and the other three sound, but should this be the case we should be inclined to adopt conservative treatment and endeavor to save the tooth.

Now since six-year olds are so pathologically liable to decay, we shall find it at all times an exceedingly difficult matter to put a permanent filling into these teeth. We should bear this fact prominently in mind when considering our treatment. Should our filling fail shortly after insertion, we shall be worse off than ever.

Hence we should undoubetdly advise extraction of all four six-year olds should approximal decay have attacked them, and should we not feel absolutely certain of introducing a permanent filling.

As a rule the wisdom teeth, from the fact that they are placed so far back in the dental arch, are of practically little or no use in mastication. Should, however, the treatment of extraction have been adopted, they will come forward together with the second molars and take up to a large extent their normal functional use in the trituration of food. In no case, however, will they be as serviceable as the six-year olds, should the latter have been preserved.

We have yet to briefly discuss our general treatment of the six-year old and the time at which it should be extracted, presuming this course of treatment has been chosen.

Only in exceptional circumstances should we extract the first permanent molars before our patient is from eleven and a-half to twelve years old, and should they be carious, we must patch them up or treat them in the ordinary way in order that they may last until the age specified. For stopping purposes, Tullivan's amalgam certainly stands *facile princeps*, as it appears to exert a hardening influence in the dentine. Should the pulp have to be drilled, great care must be taken with our drills, as the apical foramina are necessarily large.

Presuming then our patient has arrived at the age of twelve, the question arises, should we extract the six-year olds just as the second permanent molars are appearing through the gum, or should we wait until the latter are fully erupted? I think the time should depend on circumstances. Should our extraction be performed on account of the unhealthiness of the six-year olds, it should be proceeded with just before the eruption of the second molars, so that the latter may come well forward. Should there be much irregularity, however, and a good space is required, we must defer our operation until the second molars are fully erupted.—*The Dental Record.*

